# Parotid Quadrantectomy Is a Safe Management for Localized Pleomorphic Adenoma

**DOI:** 10.3389/fsurg.2018.00003

**Published:** 2018-02-05

**Authors:** Osama Hussein, Khaled Abdel Wahab, Omar Hamdy, Mohammad Arafa, Emad-Eldeen Hamed, Shady Awny, Sameh Roshdy, Adel Denewer, Mahmoud Mosbah

**Affiliations:** ^1^Surgical Oncology Department, Mansoura University Faculty of Medicine, Mansoura University Cancer Center, Mansoura, Egypt; ^2^Pathology Department, Mansoura University Faculty of Medicine, Mansoura, Egypt

**Keywords:** pleomorphic adenoma, parotidectomy, quadrantectomy, parotid gland, facial nerve

## Abstract

**Aim:**

Pleomorphic adenoma is the most common benign tumor of the parotid gland and is classically treated with superficial or total parotidectomy. Less radical surgeries have been proposed to minimize the risk of facial nerve injury. The oncological safety of these procedures remains controversial. We conducted this study to evaluate the safety of superficial hemi-lobectomy (quadrantectomy).

**Patients and methods:**

Retrospective analysis was conducted on the paraffin sections of archived superficial parotidectomy specimens from 11 male and 6 female patients (median age 33 years). The microscopic extent of extra-capsular extension was determined on pathological revision. In addition, prospective evaluation of 12 quadrantectomy procedures (M/F = 7/5, median age = 36 years) compared to 24 radical surgeries (M = F, median age = 40 years) regarding temporary and persistent facial nerve dysfunction on routine clinical assessment and recurrence rate.

**Results:**

On retrospective pathological revision, pleomorphic adenomata had a median microscopic spread of 3 mm beyond capsule in paraffin sections (SD = 3.6). On prospective analysis with a median follow-up of 33 months (range = 18–54 months), quadrantectomy had similar relative risk of temporary facial nerve dysfunction evaluated at the immediate postoperative period as well as persistent nerve dysfunction assessed at 3 months (*P* = 0.701 and *P* = 0.902, respectively). Of the whole study population, one case of recurrence after total parotidectomy was observed at mid-term follow-up (*P* = 1.000).

**Conclusion:**

Parotid quadrantectomy is a safe management for smaller pleomorphic adenomata localized close to one of the two divisions of the facial nerve.

## Introduction

Pleomorphic adenoma (PA) is the most common benign tumor of the parotid and it accounts for almost 50% of all parotid tumors. Histologically, PA is characterized by pleomorphism and contains epithelial elements mixed with various mesenchymal tissues (1). It may recur after surgery due to the microscopic extra-capsular extension of the tumor. Tumor enucleation was associated with unacceptably high 79% risk of residual disease (2) and resulted in a high rate of local recurrence which is often multifocal (1).

Malignant transformation is a known risk of PA (carcinoma ex-pleomorphic adenoma). The risk of malignancy is higher with neglected and recurrent tumors. Carcinoma ex-pleomorphic adenoma may develop in as high as 16% of recurrent cases (1).

Superficial parotidectomy (SP) has become the standard treatment for PA with a recurrence rate of 0–5%. SP, however, failed to prove an “en-bloc” operation that maintains a pre-defined safety margin. A significant proportion of planned SP terminates in a partial enucleation due to deficiency of the surgical safety margin at the area closest to the nerve branch. Partial capsular exposure has been consistently reported in studies examining the pathological parotidectomy specimens of PA. In fact, Witt has reported a 100% incidence of tumor capsule exposure after partial superficial parotidectomy (PSP) and 95% after total parotidectomy diagnosed at histopathological examination (3). Moreover, histologically positive margin(s) occurs in about 25% of SP specimens (3) and close margin occurs in about 50% of cases (4) regardless of the type of surgery. This margin positivity rate is 10 times the clinical recurrence rate of PA, which makes the importance of radical surgery questionable (4–6).

Facial nerve dysfunction is the most common complication of parotid surgery and may have a devastating sequel secondary to corneal ulceration. Facial nerve function progressively recovers with long-term follow-up and a 4% permanent nerve dysfunction can be expected after the first postoperative year (7, 8). In attempts to minimize the incidence of facial dysfunction, parotid surgeons showed renewed interest in conservative resection of PA. Although enucleation remains universally prohibited, extra-capsular dissection (ECD) (5, 6, 9, 10) and PSP (11, 12) are two widely practiced procedures. Both techniques maintain a surgical safety margin around the tumor. In ECD, the extra-capsular safety margin is not standardized and almost never exceeds 2–3 mm. on the other hand, PSP entails a generous safety margin of 2 cm. Although these two procedures gained wide acceptance among parotid surgeons, their oncologic and functional outcomes, however, remain controversial.

In view of this uncertainty, we revisited the archived pathological specimens of pleomorphic adenomata in order to characterize their microscopic extra-capsular extent and, thus, determine the optimal safety margin required for adenoma excision. Based on this study, we further proposed quadrantectomy; the standard excision of one-half of the superficial parotid lobe corresponding to one facial nerve division. This new modification of PSP starts as standard SP to the point of identification and skeletonization of the facial nerve trunk. From this point, nerve exposure will be limited to one primary division and its branches. The corresponding half of the superficial lobe will serve as the surgical margin around the tumor. The other division of the facial nerve will not be exposed. Quadrantectomy is a PSP with standard anatomical landmarks.

Since PA recurrence is a rather rare and remote complication of parotidectomy (13), our primary outcome was the incidence and prognosis of postoperative facial nerve dysfunction as a clinically more relevant parameter.

## Patients and Methods

Phase I consisted of a retrospective analysis of available records to examine archived specimens of SP resections harboring PAs. The aim of this part was to estimate the approximate microscopic extent of the neoplastic cells away from the grossly palpable tumor. The pathological revision included reevaluation of available slides and additional sectioning of the available paraffin blocks to include circumferential evaluation of the tumor capsule. Cytology materials were not revised at this stage.

**Figure 1:**
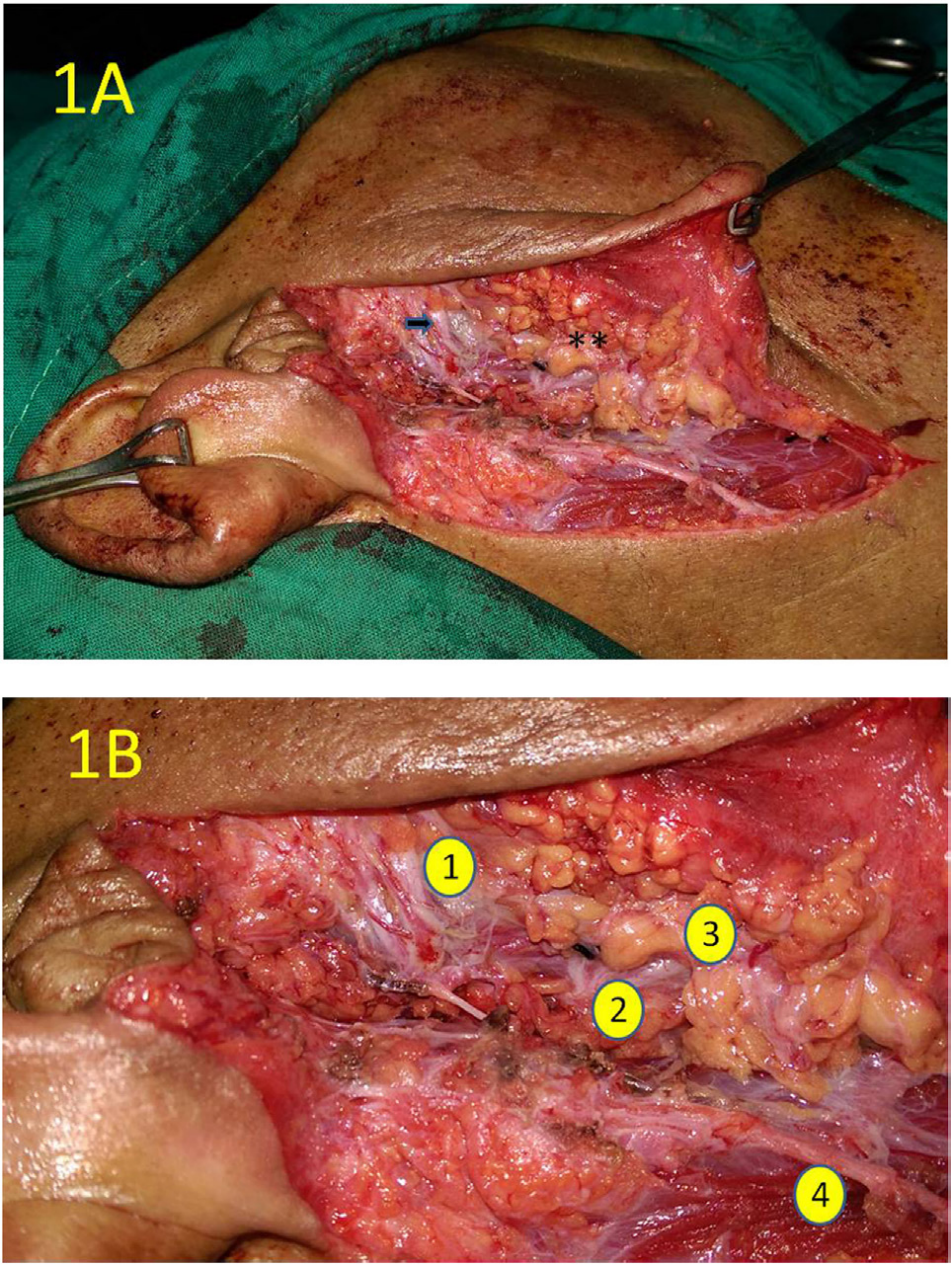
The intraoperative photograph of right parotidectomy at the completion of upper quadrantectomy. **(A)** The zygomatic branch of the facial is skeletonized “arrow.” The transected surface of the lower gland quadrant is marked by the double asterix. **(B)** Close-up view of the same operative field as **(A)** showing the zygomatic branch of the upper trunk of the facial nerve (1), the origin of the lower trunk of the facial nerve (2), the transected surface of the lower quadrant of the gland (3), and the great auricular nerve (4).

**Figure 2:**
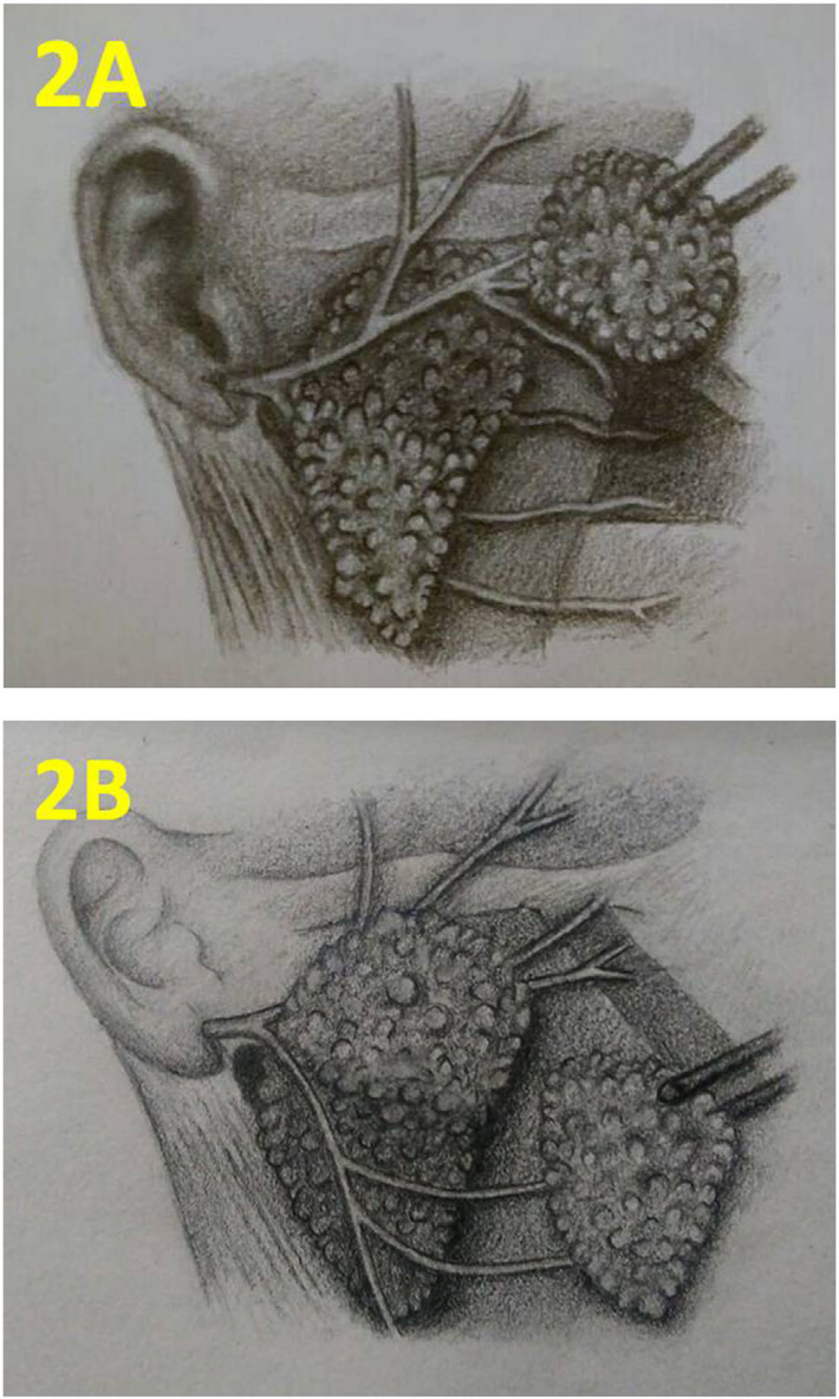
Schematic presentation of the technique. **(A)** Upper quadrantectomy. **(B)** Lower quadrantectomy. Courtesy of Manar Adel; with kind permission.

Phase II was a prospective, controlled trial comparing quadrant parotidectomy (QP) versus SP. The trial registration number: NCT01607866 (www.clinicaltrials.gov). The study included all consecutive patients with cytology proven PA of the parotid gland operated at Mansoura University Cancer Center from December 2012 till March 2016. All patients provided informed consent for participation in the trial. This study was carried out in accordance with the recommendations of the Ethics Committee of Mansoura University Faculty of Medicine guidelines, with written informed consent from all subjects. All subjects gave written informed consent in accordance with the Declaration of Helsinki. The protocol was approved by the Ethics Committee of Mansoura University Faculty of Medicine (code R/89). Patients were assigned to either QP (study group) or SP, near total, or total parotidectomy (control group). For quadrantectomy, standard incision and facial nerve trunk exposure was used. One primary division (upper or lower) was dissected free from the corresponding superficial lobe quadrant containing the adenoma. The other primary nerve division was identified at its origin but not dissected (Figures 1 and 2). Temporary facial nerve dysfunction was assessed at day 1 (temporary dysfunction) and at follow-up of at least 3 months (persistent dysfunction). Recurrence at the last available visit was recorded. Baseline criteria of the experimental versus control group were compared using Mann–Whitney test for continuous variables. Categorical outcome data were analyzed using Fisher exact test and relative risk estimation.

## Results

Retrospective pathological revision of seventeen SP specimens was performed. This part of the study included the parotidectomy specimens of eleven males and six females. Median age was 33 years (range = 13–60) and median tumor size was 2.5 cm (range = 1.5–6.0). Pathological revision defined the maximal limit of extra-capsular tumor by 10 millimeters (median = 3 mm, Mean = 4.05 mm, SD = 3.6, range = 1–10 mm). The measurements in paraffin sections underestimate the distance in living tissue due to universal shrinking of excised specimens. Living tissue measurements are assumed to be 1.5–2 times those of the archived materials.

The prospective trial was terminated due to limited accrual. Eligible patients were excluded from randomization due to one or more factors. Physician-initiated exclusion was mainly due to intermediary tumor position that would not fit into neither superior nor inferior quadrantectomy or due to large tumor size that was judged a relative contraindication by the treating surgeon. Other eligible patients refused randomization. Eventually, the data of 12 quadrantectomy procedures were available for analysis; of them 9 patients were non-randomly elected.

The 12 cases of QP were compared to 24 radical operations (superficial, near total, or total parotidectomy). The quadrantectomy group included seven males and five females and the control group included equal numbers of both genders. Median tumor size was 2.8 cm in QP versus 2.9 cm in control group (*P* = 0.650). Median age of the patients at presentation was 36 and 40 years for the quadrantectomy and radical groups, respectively (*P* = 0.347). Median follow-up period was 33 months (range 18–54) and was comparable in both groups (31.5 months for quadrantectomy patients versus 33 for control group; *P* = 0.233).

Quadrantectomy involved upper facial nerve trunk in four operations and lower trunk in eight surgeries.

Temporary facial dysfunction was observed in 16.7 and 29.2% of quadrantectomy and radical surgery, respectively (*P* = 0.701). Permanent facial dysfunction occurred in 8.3 and 16.7% of QP and radical surgery, respectively (*P* = 0.902). In comparison to quadrantectomy, radical surgery had a relative risk of 1.18 and 1.10 for temporary and persistent facial dysfunction, respectively (Table 1). The control group of radical surgery included 17 SP operations in addition to three near total and four total parotid gland excision as will be discussed in the following paragraphs.

**Table 1:** Treatment outcome of the prospective part of the study.

Treatment outcome		Study group (*n* = 12) (quadrant parotidectomy)	Control group (*n* = 24) (classic parotidectomy)	*P*-value
Temporary dysfunction	Number (%)	2 (16.7%)	7 (29.2%) Relative risk (95% confidence interval) = 1.18 (0.82–1.7)	0.701

Permanent dysfunction	Number (%)	1 (8.3%)	4 (16.7%) Relative risk (95% confidence interval) = 1.10 (0.86–1.41)	0.902

Recurrence	Number (%)	0 (0%)	1 (4.2%) Relative risk (95% confidence interval) = 1.04 (0.96–1.13)	1.0000

Superficial parotidectomy resulted in temporary nerve dysfunction in 29.4% and persistent dysfunction in 17.6%; which was not significantly different from quadrantectomy (*P* = 0.665 and *P* = 0.662, respectively). In comparison to quadrantectomy, SP had a relative risk of 1.81 and 1.10 for temporary and permanent facial dysfunction, respectively.

The intraoperative assessment resulted in extending the extent of surgery to total/near total parotidectomy due to the extent of the adenoma to the deep lobe. Total/near total parotidectomy resulted in temporary nerve dysfunction in 28.6% and permanent dysfunction in 14.3%; which was not significantly different from quadrantectomy (*P* = 0.603 and *P* = 1.000, respectively). In comparison to quadrantectomy, total/near total parotidectomy had a relative risk of 1.17 and 0.76 for temporary and persistent facial dysfunction, respectively.

A female, 57 years patient developed carcinoma ex-pleomorphic adenoma 4 months after total parotidectomy for a PA. The recurrent mass has occupied the pre-styloid and post-styloid parapharyngeal spaces and has metastasized to both lungs. The patient received cisplatin/5-fluorouracil and palliative external-beam irradiation. She had stationary course for 26 months complicated by pulmonary embolism. At the time of writing, the patient has experienced progression on chemotherapy. This case represents a recurrence rate of 14.3% after total/near total parotidectomy and a rate of 4.2% in the control group as a whole. There were no incidents of tumor recurrence after quadrantectomy or after SP. The recurrence rate was not significantly different in quadrantectomy versus total/near total parotidectomy or versus all control group patients (*P* = 0.400 and *P* = 1.000, respectively).

## Discussion

In this study, we characterized the microscopic extra-capsular tumor spread of PA. Extra-capsular pseudopodia and satellites are notorious features of PA and earn the tumor its characteristic reputation for liability to recur. Although capsular thickness is well described, the measurement of maximal extra-capsular tumor extent has been hitherto poorly characterized (1). We examined archived material of PA for the maximal extra-capsular spread and defined it as 10 mm in fixed specimens. The findings of this histological study stand against the justification of conservative surgery of PA. Based on retrospective studies, recent metanalyses (6, 10) showed comparable recurrence rates of ECD and SP. However, recurrence of PA is rather rare event that occurs several years after resection. Zernial and colleagues failed to detect any single event of recurrence in 43 patients followed for 2–20 years (median of 8.1 years) (13). Zbären and Stauffer reported two events of recurrence 5 and 8 years after resection in a cohort of 218 patients with a median follow-up of 9.6 years (14) accounting for a recurrence rate of 0.9%. The retrospective analysis by Koch and his colleagues included 458 total and SPs and their recurrence rate was 2.2% after a median follow-up of 6 years (15). In view of this peculiar behavior, it appears that most of the available studies are probably underpowered to detect a difference in recurrence rate between ECD and SP. Our observation of microscopic adenoma extension 1–10 mm beyond their capsule in fixed specimens stands as a clear evidence against the adequacy of ECD with its margin of a few millimeters. On the other hand, the generous safety margin of PSP adequately encompasses almost all possible microscopic tumors. An extremely favorable recurrence rate of 0–0.7% has been reported after PSP (15, 16). Standard nerve exposure is, however, omitted in PSP which may increase the risk of injury. We proposed QP as a standardized controlled partial parotidectomy. Quadrantectomy entails dissection of only one of the two primary divisions of the facial nerve. QP is a procedure of standardized anatomical dissection comparable to and equivalent to superficial hemi-lobectomy. One facial nerve division (either the upper zygomatico-temporal or the lower cervico-mandibular trunk) is dissected and secured under vision in a standard way and the other division is kept out of the surgical field. The main advantage of QP over other conservative procedures is the proper identification of nerve structures with the added security of avoiding blind mani-pulations.

In this study, the operative extent in seven patients scheduled for SP was expanded to total or near total parotidectomy in response to intraoperative findings. This may be attributed in part to the wide range of tumor size included in the trial. The heterogeneity of the control group represents a limitation in the study although sub-group analysis did not change the study conclusions. Our experience showed that the scope of parotid quadrantectomy is limited to smaller tumors with favorable location close to one division of the facial nerve.

Early termination due to lack of accrual was another limitation of this study. In view of our results, our study proved a useful safety study that can be followed by a larger controlled trial, preferably with pre-randomization exclusion of cases according to extremes of tumor size or possibility of total gland excision as assessed by the surgical team. This may help avoid post-randomization drop-out.

## Conclusion

Based on our histological and empirical studies, QP is a safe operation for localized smaller adenomas. The operation effectively protects the facial nerve.

## Ethics Statement

This study was carried out in accordance with the recommendations of the Ethics Committee of Mansoura University Faculty of Medicine guidelines, with written informed consent from all subjects. All subjects gave written informed consent in accordance with the Declaration of Helsinki. The protocol was approved by the Ethics Committee of Mansoura University Faculty of Medicine (code R/89).

## Author Contributions

The corresponding author OH conceived the study, designed the technique, analyzed the data, and wrote the manuscript. KW, OmH, EEH, MM, SA, and SR collected the data, MA performed the histopathological examination, and all authors contributed to the patients’ management and approved the manuscript.

## Conflict of Interest Statement

The authors declare that the research was conducted in the absence of any commercial or financial relationships that could be construed as a potential conflict of interest.
